# Cistinuria: sedimento de orina como herramienta diagnóstica

**DOI:** 10.1515/almed-2019-0031

**Published:** 2020-04-27

**Authors:** María Pierna, Mohamed Abdelgabar, Raquel Fernández-Rivas, Miguel Fernández-Burriel

**Affiliations:** Servicio de Análisis Clínicos, Hospital de Mérida, Mérida, Spain; Sección de Urgencias, Hospital de Mérida, Mérida, Spain; Servicio de Análisis Clínicos, Hospital de Mérida, Polígono Nueva Ciudad s/n. 06800, Mérida, Spain

**Keywords:** cistinuria, cristales cistina, litiasis renal, sedimento urinario

## Abstract

**Objetivos:**

Demostrar la importancia de la realización del estudio del sedimento urinario con la correcta interpretación y tipificación de los cristales como herramienta diagnóstica en el laboratorio clínico, así como de la elaboración de protocolos que determinen la necesidad de realizar este tipo de exámenes microscópicos de sedimento urinario de forma rutinaria.

**Caso clínico:**

Se trata de un paciente varón de edad avanzada sin antecedentes personales ni familiares de interés. Se presenta con dolor fijo y sin irradiar de tres días de duración en fosa iliaca izquierda, siendo la primera que vez que padece episodios de dolor de este tipo. El sistemático de orina revela proteinuria, hematuria y el sedimento muestra abundantes cristales hexagonales y planos, típicos de cistina. El análisis de aminoácidos confirma el hallazgo encontrándose concentraciones elevadas de aminoácidos aminoácidos dibásicos y de cistina.

**Conclusiónes:**

El estudio del sedimento urinario por parte del laboratorio clínico pone de manifiesto la presencia de un caso de cistinuria por la aparición en él de cristales patognomónicos de dicha patología en una edad avanzada y sin antecedentes previos. Este caso clínico tiene especial interés al demostrar la importancia del sedimento urinario como herramienta diagnóstica en la evaluación de una enfermedad genética, que se pone de manifiesto como un simple cólico nefrítico.

## Introducción

La cistinuria se engloba dentro de los trastornos genéticos conocidos como aminoacidurias. Tiene una prevalencia global de 1:7000 [1] y su alteración básica radica en un defecto del trasporte transepitelial del aminoácido cistina, y de los aminoácidos dibásicos: ornitina, lisina y arginina. Esto tiene como resultado una disminución de la absorción de estos aminoácidos en el intestino y a nivel renal. Los aminoácidos filtrados en el glomérulo son deficitariamente reabsorbidos en el túbulo renal proximal produciéndose un aumento de su concentración en la orina. Así, se favorece la sobresaturación de cistina y la consecuente formación de cristales urinarios, sobre todo a pH ácido, que pueden acabar en la formación de cálculos de cistina [2, 3].

Es la patología hereditaria más frecuente de las que afecta al transporte de aminoácidos, aunque su consecuencia clínica, la litiasis renal de cistina es uno de los tipos de enfermedad litiásica renal menos frecuente [4]. Su base genética se asocia a mutaciones en dos genes: *SLC3A1* que codifica para la subunidad pesada de un trasportador especifico de cistina y aminoácidos básicos (b(0,+)): rBAT (related b^o,+^ aminoacid transporter) y *SLC7A9* que codifica para la unidad funcional, o subunidad ligera de dicho trasportador: b(0,+)AT [2, 3].

El modo más habitual de presentación es a través de la sintomatología característica de la litiasis urinaria: hematuria microscópica y macroscópica, cólico nefrítico con o sin expulsión de cálculo y dolor lumbar. Este conjunto de síntomas suelen ser muy recurrentes y pueden ir acompañados de infección urinaria, obstrucción de las vías urinarias y, ocasionalmente, de fallo renal [5]. Los cálculos exclusivos de cistina son de color amarillento, de brillo perlado y aunque radiopacos en relación con las moléculas de sulfuro que contiene y su densidad, la radiografía de abdomen es poco sensible para detectarlos y se observan solamente en el 50% de los pacientes afectados [6].

El análisis del sedimento urinario nos puede ayudar a la tipificación de las litiasis a través de sus cristales, determinando como en el caso de la cistinuria, un diagnóstico clínico. Los cristales de cistina, son patognomónicos de la cistinuria, apareciendo como estructuras hexagonales y generalmente planos (en ocasiones, especialmente en fases de actividad pueden alcanzar un grosor de 1 mm). Así, el caso clínico que se presenta a continuación tiene especial interés desde el punto de vista del laboratorio clínico, al demostrar la importancia del sedimento urinario como herramienta diagnóstica en la evaluación de una enfermedad genética con aparición por primera vez en edad adulta, que puede pasar desapercibida en una primera valoración.

## Caso clínico

Se trata de un paciente varón de 49 años de edad sin antecedentes personales ni familiares de interés, y sin ningún tratamiento pautado. El paciente fue valorado por el Servicio de Urgencias de un hospital dentro de su comunidad de residencia donde se le administró metamizol y metoclopramida intramuscular para alivio del dolor. Transcurridas 48 horas acudió al Servicio de Urgencias de nuestro hospital refiriendo dolor fijo y sin irradiar de tres días de duración en fosa iliaca izquierda, con puño percusión renal negativa bilateral. El paciente comentó que es la primera vez que padecía episodios de dolor de este tipo. No mostraba disuria, ni urgencia urinaria, aunque si polaquiuria.

**Figura 1:**
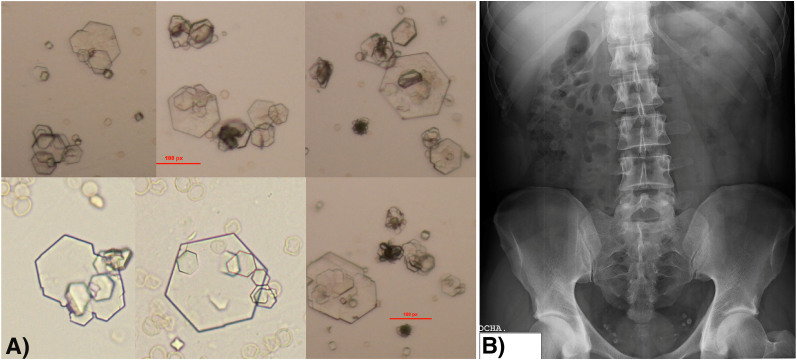
Imágenes de algunas de las pruebas complementarias que se realizaron al paciente. (A) Sedimento urinario al ingreso del paciente referido (400×). Se observan cristales hexagonales y planos de pequeño tamaño (10–20 µm) con diferentes niveles de maclación. En las distintas imágenes aparecen maclas de mediano tamaño y/o de gran tamaño compuestas en estratos o escalera característica de este tipo de cristales. (B) Radiografía simple de abdomen mostrando varios flebolitos no relacionados con la litiasis.

La exploración física fue normal, y los resultados de la analítica sérica mostraron como único dato significativo una concentración de proteína C reactiva (PCR) de 12 mg/l (VR: 0–6 mg/l), manteniéndose el resto de la magnitudes bioquímicas dentro de los intervalos de referencia (Tabla 1A). Sin embargo, el análisis automatizado mediante citometría de flujo (UF-1000i, Sysmex) y tira urinaria (COBAS U411) de la orina de micción única reveló un pH de 5, con proteinuria, hematuria y leucocituria (Tabla 1B). En el sedimento de la misma se observaron abundantes cristales hexagonales y planos, típicos de cistina (Figura 1A). En base a estos resultados, se analizó la orina mediante HPLC (Biochrom 30Plus Amino Acid Analyzer) en el Servicio de Metabolopatías del Complejo Hospitalario Universitario de Badajoz encontrándose concentraciones elevadas de aminoácidos dibásicos (ornitina, lisina y arginina), así como de cistina (Tabla 1B). Esto confirmaría la sospecha clínica, ya que debido a la falta de reactivos, no se pudieron realizar los test de Brand, ni el método abreviado descrito anteriormente por nuestro equipo [7]. En la radiografía abdominal no se pudieron objetivar cristales, encontrándose tan solo algunos flebolitos a nivel pélvico sin aparente relación con la patología del paciente (Figura 1B).

**Tabla 1:** Resultados de la analítica del paciente.

A) Bioquímica		
Parámetro	Resultado	VR
Glucosa, mg/dL	76	65–110
Urea, mg/dL	43	10–50
Creatinina, mg/dL	1.26	0.60–1.40
Sodio, mmol/L	138	135–153
Potasio, mmol/L	3.9	3.5–5.3
Bilirrubina total, mg/dL	1.0	0.1–1.2
LDH, U/L	173	135–225
GPT, U/L	18	5–40
α-Amilasa, U/L	44	10–100
PCR, mg/dL	12	0–6

B) Análisis de orina		
Citometría de flujo, tira de orina y sedimento urinario		
Leucocitos, μL	36	0–25
Hematies, μL	582	0–25
Cilindros hialinos, μL	1.1	0.0–3.0
Células epiteliales, μL	5	0–50
Bacterias, μL	22	0–10,000
Proteinas, mg	27	0–20
pH	5	
Sedimento urinario	Abundantes cristales de Cistina	

Análisis de aminoácidos		
Arginina, mmol/mol creat.	7.80	1–7
Lisina, mmol/mol creat.	156.79	12–52
Cistina, mmol/mol creat.	23.66	1–19
Omotina, mmol/mol creat.	26.01	0–5

(A) Bioquímica sérica. (B) Análisis automatizado mediante citometría de flujo, tira de orina, sedimento urinario y los resultados más relevantes para el caso del análisis de aminoácidos. Creat, creatinina; VR, valores de referencia.

Se pautó como tratamiento abundante hidratación y metamizol (575 mg) cada 8 horas durante 3–5 días recomendándole acudir a su Área de Salud de referencia para la confirmación del diagnóstico, control y seguimiento.

## Discusión

Ante los hallazgos encontrados y en correlación a la sintomatología se consideró que el paciente presentaba un cólico renal no complicado secundario a cistinuria. Al ser un paciente transeúnte, perteneciente a otra comunidad autónoma, tras el tratamiento sintomático del dolor se procedió a cursar el alta hospitalaria con una pauta de abundante hidratación y analgésicos. Se le recomendó que acudiese a su Área de Salud de referencia para la confirmación del diagnóstico mediante la determinación de la composición exacta de los cálculos (si los expulsase), determinación de los niveles de cistina en orina de 24 horas, estudio genético para la caracterización del gen responsable, así como un correcto tratamiento y seguimiento de la patología. La eliminación de los cálculos de cistina por litotricia es difícil debido a su extrema dureza y a su alta tasa de recidiva. Así, se aconseja una terapia no invasiva basada en una alta ingesta de líquidos, alcalinización de la orina con citrato de potasio o acetazolamida, y empleo de agentes quelantes como D-penicilamina, tiopronina (*α*-mercaptopropionilglicina, *α*-MPG), bucilamina, o el captopril que disminuyan la concentración de cistina libre, y forman compuestos derivados más solubles que son fácilmente eliminados por la orina [8].

El cólico nefrítico es uno de los motivos más frecuentes de consulta en los Servicios de Urgencias, sobre todo en épocas estivales y en el 90 % de los casos a la litiasis renoureteral aguda, parcial o completa [9]. Es importante tener en cuenta que es un diagnóstico clínico en el que atribuyen un conjunto de síntomas y signos a una obstrucción aguda del tracto urinario superior de diversa etiología (litiasis, coágulos, etc.). Por lo que es fundamental, además de la clínica, realizar una exploración física, un diagnóstico diferencial, ya que como muchas otras entidades puede tener presentaciones atípicas, y exploraciones complementarias básicas como una radiografía simple de abdomen y un sedimento urinario por parte del laboratorio clínico. Así, debido a la edad del paciente y al no tener antecedes personales ni familiares de interés el diagnóstico diferencial estuvo enfocado hacia la litiasis urinaria.

Después de realizar una analítica que incluía bioquímica, hemograma y análisis de orina, los datos más llamativos fueron el aumento de la concentración sérica de PCR, aumento de las proteínas en orina y una clara hematuria característica, como se describe anteriormente, de una litiasis urinaria. Sin embargo, el hallazgo más significativo para determinar el diagnóstico fue la aparición de cristales hexagonales en el sedimento urinario. Este tipo de cristales tienen significación clínica diagnostica por sí mismos, su presencia en la orina es la expresión de un exceso de eliminación urinaria de este aminoácido y patognomónico de esta enfermedad [3]. El análisis de aminoácidos urinarios determinó un aumento anormal de las concentraciones de aminoácidos dibásicos y cistina en la orina confirmando este hallazgo. La radiografía simple de abdomen no mostro ninguna imagen sugerente de litiasis, quizás debido a su limitada sensibilidad para detectar este tipo de cálculos [6].

El diagnóstico de sospecha de cistinuria se debe implantar en todo paciente con cálculos urinarios o con sintomatología urinaria, en particular cuando aparece a edades tempranas, existe una tasa de recurrencias elevada, y se presupone asociación familiar. Como en los pacientes con otros trastornos monogénicos asociados con la formación de cálculos, la sintomatología suele ser precoz, comenzando la formación de los cálculos a una edad joven y siendo su diagnóstico excepcional, como en este caso, en una edad avanzada [10]. El riesgo de formación de cálculos en la cistinuria a lo largo de la vida es superior al 50% y su frecuencia de formación varía entre escasas veces a lo largo de la vida en las formas leves, hasta varias veces por año en las formas más graves. Si bien, no todos los pacientes cistinúricos desarrollan litiasis, pudiendo estar su formación influenciada por diversos factores como el balance salino, la excreción de inhibidores de la calculogénesis o componentes ambientales [11]. Así, se recomienda el estudio del sedimento urinario en todo paciente con cólico renal, independientemente de la edad y ante la ausencia de episodios anteriores, ya que supone una prueba simple, no invasiva y barata que nos puede llevar a hallazgos como el descrito aquí en los que su aparición resulta inusual y puede pasar desapercibida.

## Conclusiones

Aunque lo sufrido por el paciente es claramente una litiasis urinaria, el estudio del sedimento urinario por parte del laboratorio clínico pone de manifiesto la presencia de un caso de cistinuria por la aparición en él de cristales patognomónicos de dicha patología en una edad avanzada y sin antecedentes previos. Si bien, este hallazgo debe ser acompañado de un protocolo diagnóstico de confirmación que incluya cuantificación de los aminoácidos urinarios, análisis de los cálculos, determinación de la concentración de cistina en orina de 24 horas y estudio genético para la caracterización del gen responsable.

Este caso clínico revela la importancia de la realización del estudio del sedimento urinario con la correcta interpretación y tipificación de los cristales como herramienta diagnóstica en el laboratorio clínico, así como de la elaboración de protocolos que determinen la necesidad de realizar este tipo de exámenes microscópicos de sedimento urinario de forma rutinaria.
